# Plant NETWORKED and VAP27 Proteins Work in Complexes to Regulate Membrane-Based Functions

**DOI:** 10.1177/25152564251342533

**Published:** 2025-05-27

**Authors:** Patrick J. Duckney, Pengwei Wang, Patrick J. Hussey

**Affiliations:** 1 Department of Biosciences, 3057 Durham University, Durham, UK; 2 98464 Health and Life Sciences, Oxford Brookes University, Oxford, UK; 3 Key Laboratory of Horticultural Plant Biology, College of Horticulture and Forestry Sciences, Huazhong Agricultural University, Wuhan, China

**Keywords:** NET, VAP27, actin, cytoskeleton, membrane, contact site, organelle, plant, endoplasmic reticulum, autophagy

## Abstract

Eukaryotic cells are subdivided into specialised organelle compartments, each with unique physiological environments and functions. Interaction and cross-talk between organelles is inherent to Eukaryotic life, and each organelle is physically interconnected to their surrounding subcellular components including the cytoskeleton and adjacent membrane compartments. In animals and yeast, the mechanisms of organelle interaction have been well characterised and are known to have fundamental importance to life. In contrast, we are only beginning to understand the mechanisms and functions of such interactions in plants. The discovery and ongoing characterisation of the NETWORKED (NET) protein family of plant actin-membrane adaptors has greatly advanced our understanding of the mechanisms of organelle-cytoskeletal interaction. Furthermore, unfolding investigation into the NET proteins has revealed their binding partner, VAMP-ASSOCIATED PROTEIN-27 (VAP27), to be a regulator of organelle tethering and interaction with previously unknown, specialised roles in plants. Research on NET and VAP27 proteins has rapidly increased our knowledge of the mechanisms regulating membrane interaction in plants, their functions in regulating cell structure and organisation, as well as their importance to plant growth, development and stress-response. Here, we discuss the discovery and characterisation of the NET and VAP27 proteins, their regulation of organelle interaction and their functions in plants.

## Introduction

The actin cytoskeleton is crucial to Eukaryotic life, with fundamental importance in dynamic cell processes including cell growth, division, motility and stress-response. At the subcellular level, the functions of the actin cytoskeleton include regulation of organelle transport, structuring and remodelling through the generation of physical force upon organelle membranes (Wang et al., 2015). Physical connection between filamentous actin (F-actin) and cell membranes therefore has implicit importance to a plethora of plant life processes. In contrast to animal and fungal models, the mechanisms of actin-membrane interactions in plants had long presented a gap in our knowledge (Deeks et al., 2012). Well characterised mammalian proteins that are known to aid in the linkage of actin to cell membranes (for example, ɑ-Actinin and Spectrin) are not conserved in plants, and the identities and functions of their plant counterparts have remained enigmatic. With a basic cell structure that is highly distinct from animals and fungal organisms, the specific functions of the plant cytoskeleton are likewise distinct, as are the molecular mechanisms that regulate them (Lloyd, 2012). It is therefore to be expected that plants have evolved unique actin-regulatory proteins to drive plant-specific cell processes, and plant-specific mechanisms of actin-membrane interaction.

The discovery of the plant-specific NETWORKED (NET) superfamily of actin-organelle linker proteins has advanced our understanding of the molecular mechanisms of plant actin-membrane interactions and their importance to cell structure, function and signalling. As discussed in this review, the NET proteins have been shown to regulate cytoskeletal organisation, trafficking, membrane remodelling, cell wall deposition and cell responses to biotic and abiotic stress. Notably, unfolding research on the NET family has revealed their involvement in the regulation of organelle membrane contact sites; specific sites of physical connection between individual organelle compartments with fundamental importance to organelle structure and signal exchange (Scorrano et al., 2019; Duckney et al., 2022. Voeltz et al., 2024). Here, the recent discovery of the NET-interacting protein, VAP27/VAP (VAMP ASSOCIATED PROTEIN-27; Wang et al., 2014), has shown it to be an important regulator of organelle tethering at contact sites between various organelles with importance to a plethora of cell functions including cell growth, stress-response, organelle structure, cytoskeletal regulation, cell wall deposition, autophagy, metabolism and trafficking, as will be discussed below.

Here we discuss current research advances on the NET and VAP27 protein families, and their demonstrated roles in the regulation of organelle structure and function. Outside of the NET protein family, there are few other plant-specific proteins that are known to link actin to membranes (the only example being the actin-chloroplast linker, CHLOROPLAST UNUSUAL POSITIONING; CHUP1, Oikawa et al., 2003, Schmidt von Braun and Schlieff, 2008). This review will focus on current research on the NET protein family, that comprises a large number of plant-specific actin-membrane linkers with diverse roles, which has expanded our understanding of a plethora of mechanisms underpinning plant cell structure and function. Similarly, VAP27 proteins are one of several classes of membrane tethering proteins, including plant SYNAPTOTAGMIN (SYT; Siao et al., 2016; Lee et al., 2019) proteins and MULTIPLE C2 DOMAINS AND TRANSMEMBRANE DOMAIN PROTEINS (MCTP; Pérez-Sancho et al., 2024), however this review will focus on VAP27 due to the comparatively extensive research on the multifaceted roles of this protein family at multiple organelle contact sites. Together, ongoing research on the NET and VAP27 proteins represent a continuous strand of research that has greatly advanced the field of plant cell biology.

## The NETWORKED Superfamily of Plant Actin-Membrane Linker Proteins

The Arabidopsis NET family comprises thirteen proteins that are defined by their unique NET Actin-Binding (NAB) domain. This plant-specific actin-binding domain interacts directly with filamentous actin and is necessary for the localisation and interaction of NET proteins with the actin cytoskeleton *in vivo* (Deeks et al., 2012). The NAB domain was discovered in a high-throughput protein subcellular localisation screen in which libraries of Arabidopsis cDNA-GFP fusions were expressed *in planta* and their localisations were characterised using confocal microscopy (Escobar et al., 2003). In this screen, a cDNA fragment encoding NET1A^1−288^-GFP decorated the actin cytoskeleton and was found to contain a novel plant specific actin-binding domain comprising NET1A^1−94^. This NAB domain was found to be conserved in an additional 12 previously unidentified Arabidopsis proteins, which can be subdivided into four distinct subfamilies (NET1, NET2, NET3 and NET4) based on sequence homology and expression pattern (Deeks et al., 2012). Crucially, it was observed that members of each subfamily localise to distinct organelle membranes *in vivo*, connecting them to the actin cytoskeleton. Subsequent analysis of each NET protein subclade has revealed novel mechanisms of actin-membrane interaction in plants, and the importance of the actin cytoskeleton in the regulation of plant organelle structure and function.

**Figure 1.**
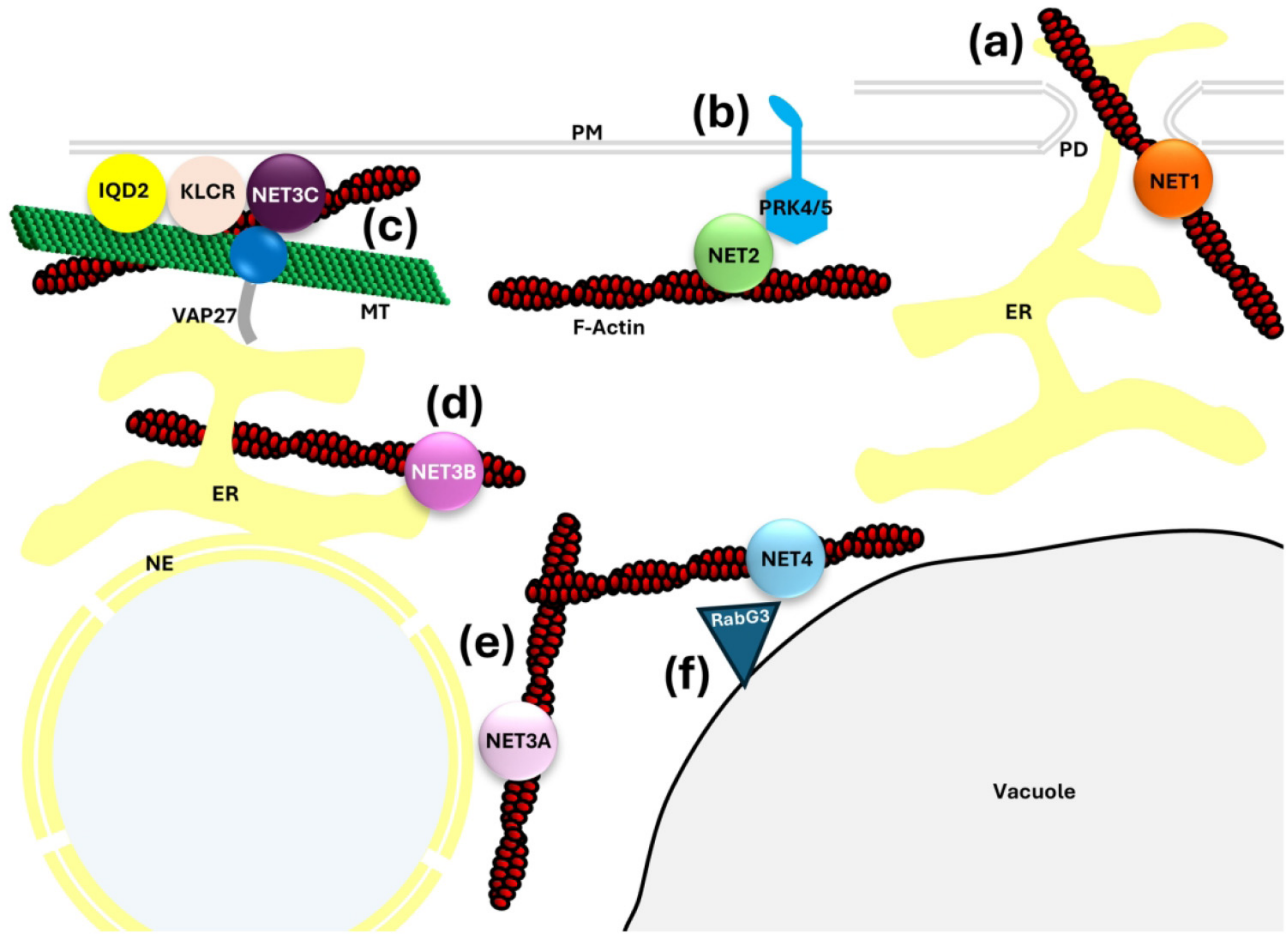
Plant-Specific NETWORKED Proteins Mediate Actin-Membrane Interactions and Dynamic Cell Organisation. (a). NET1-family proteins bind actin at the plasmodesmatal (PD) pore. NET1 proteins localise to PD and bind actin. (b). NET2 family proteins regulate actin-PM interactions and actin organisation. NET2-family proteins localise to the PM through interaction with membrane integral PRK4 and PRK5, and bind longitudinally orientated actin cables. NET2 proteins anchor and organise actin cables at the PM parallel to polar cell expansion, facilitating polarised transport in growing pollen tubes. (c - e). NET3-family proteins connect actin to the ER. (c). NET3C forms an EPCS-actin-microtubule (MT) nexus. NET3C localises to the PM through a c-terminal lipid-binding motif and interacts with ER-integral VAP27 to form EPCS. NET3C binds actin through its N-terminal NAB domain, and interacts with microtubule-binding KLRC-family proteins and IQD2. Together, this complex organises the cortical microtubule, actin and ER networks. (d). NET3B tethers ER tubule networks to actin filaments. (e). NET3A localises to the nuclear envelope (NE) with yet-unknown functions. (f). NET4-family proteins regulate actin-tonoplast interaction. NET4A and NET4B bind F-actin and localise to the tonoplast through interaction with RabG3-family small signalling GTPases. NET4 proteins regulate dynamic, actin-driven reorganisation of the tonoplast in response to extracellular signals under control of RabG3 signalling cascades.

The first member of the NET superfamily to be characterised was NET1A; one of four proteins in the NET1 subclade (comprising NET1A, NET1B, NET1C and NET1D). The NET1-family proteins are hypothesised to be involved in cell-to-cell communication through the plasmodesmatal pore; nanoscopic membrane channels establishing ‘communication bridges’ between the cytosol of neighbouring cells (Pérez-Sancho et al., 2024). NET1A localises to the plasmodesmata, binds F-actin through its NAB domain (Figure 1a) and is important in controlling root growth, as *net1a/net1b* double mutants exhibit defects in normal root cell expansion (Deeks et al., 2012). The plasmodesmata is a dynamic structure in which the diameter of the pore is tightly regulated to control the flux of signalling molecules between cells (Tee and Faulkner, 2024). The actin cytoskeleton appears to be an important regulator of plasmodesmatal pore diameter and transport (Diao and Huang, 2021). Therefore, with roles in mediating actin-membrane connection, it is plausible that NET1-family proteins could be implicated in regulating actin-dependent plasmodesmatal function. However, a mechanism by which NET1-family proteins connect actin to the plasmodesmata has yet to be identified: NET1 proteins possess no transmembrane domain or apparent post-translational modification that may endow direct membrane-interacting ability. NET1 membrane association is likely to occur indirectly through interaction with an intermediary binding protein which has yet to be discovered.

Actin-plasma membrane (PM) interactions are partially regulated by the NET2 protein subfamily in Arabidopsis, with emerging roles in the regulation of cell polarity through the anchorage and organisation of cortical actin cables. The NET2 subfamily comprises NET2A, NET2B, NET2C and NET2D, which are expressed specifically in pollen (Deeks et al., 2012). Of these proteins, NET2A has been most intensively studied. NET2A associates with actin *in vivo* through its N-terminal NAB domain and localises to punctae at the pollen tube plasma membrane which are hypothesised to anchor actin cables to the cell cortex (Figure 1b; Duckney et al., 2017). Physical connection between actin filaments and the plasma membrane are formed through the interaction of NET2A with the integral membrane receptor proteins POLLEN RECEPTOR-LIKE KINASE 4 (PRK4) and PRK5 (Figure 1b; Duckney et al., 2017). These actin-plasma membrane connections are important in regulating cell polarity in growing pollen tubes: *net2* loss-of function mutants exhibit defective pollen tube directional growth resulting from uncoupling of F-actin from the cell cortex, cytoskeletal disorganisation and aberrant polar vesicle secretion (Duckney et al., 2021). Actin-membrane interactions mediated by NET2 and PRKs have interesting implications in coordinating pollen tube responses to extracellular signals during fertilisation. As upstream regulators of chemotactic pollen tube growth, it is possible that PRKs may regulate actin-driven pollen tube growth downstream of extracellular signal perception through regulation of NET2 proteins at the PM (Huang et al., 2014; Takeuchi and Higashiyama, 2016).

NET3-family proteins link actin to specific ER subdomains and serve as a nexus of cytoskeletal organisation and organelle interaction in plants (Figure 1c – e). NET3A binds F-actin through its NAB domain and localises to the nuclear envelope (Figure 1e) through an unknown mechanism, and, with redundancy to NET3C, is important for regulation of cell morphology in leaf epidermal tissue (Deeks et al.*,* 2012; Zang et al., 2021). NET3B localises to tubular ER networks through its c-terminal coiled-coil domain via an unknown mechanism, and there binds F-actin to enhance ER-actin connection (Figure 1d, Wang and Hussey, 2017). NET3B is an essential protein with functional redundancy to NET3C, and *net3b/*NET3C RNAi mutants are male-gamete lethal (Wang et al., 2014). However, the functions of NET3B in the regulation of cell structure and function still remain to be explored. Of the NET3 protein family, NET3C has been the most intensively studied. NET3C has emerged as a component of ER-PM contact sites (EPCS), at which it has key roles in promoting organelle tethering, and the organisation of the cortical actin and microtubule cytoskeletons. NET3C localises to discreet microdomains of the plasma membrane through a putative c-terminal lipid-binding domain and binds F-actin through an N-terminal NAB domain (Figure 1c). ER-PM tethering is regulated by an interaction between NET3C and the ER-integral protein VAP27. Ectopic overexpression of NET3C and VAP27 in *N. benthamiana* leaf epidermal cells results in enhanced EPCS surface area, indicating that the complex promotes organelle association (Wang et al., 2014). At EPCS, NET3C forms a complex with the microtubule-binding proteins, KINESIN LIGHT CHAIN-RELATED 1 (KLCR1; also named CELLULOSE SYNTHASE-MICROTUBLE UNCOUPLING 1; CMU1), and IQ-DOMAIN 2 (IQD2), to promote cross-linking and organisation of the actin and microtubule cytoskeletal networks, organisation of the cortical ER and EPCS structure (Figure 1c). The NET3-KLCR-IQD2 complex is important to the regulation of plant morphology: disruption of this complex results in defects in cytoskeletal organisation, likely resulting in aberrant cell wall deposition and frameworks for cell expansion (Zang et al., 2021).

NET4 proteins mediate actin-tonoplast interaction in Arabidopsis, with functions in the dynamic control of cell morphology through regulation of vacuole volume (Kaiser et al., 2019; Hawkins et al., 2023). NET4A and NET4B localise to the tonoplast and there bind actin through their NAB domains (Figure 1f). The physical link between NET4 and the tonoplast is established through interaction with the tonoplast-associated Rab7-family small signalling GTPase, RabG3, which recruits NET4 proteins to the tonoplast from the cytosol (Hawkins et al., 2023). In Arabidopsis, NET4A has functions in the regulation of vacuolar morphology and NET4A-overexpressing transgenic plants exhibit decreased vacuolar volume (Kaiser et al., 2019). The remodelling of vacuole structure by NET4 is controlled by RabG3 signalling cascades. Rab proteins serve as ‘molecular switches’ that shuttle between ‘inactive’ GDP-bound and ‘active’ GTP-bound states, which determine their localisation and binding partners (Woollard and Moore, 2008). Once activated, RabG3 relocates from the cytosol to the tonoplast and interacts with NET4. In stomatal guard cells, RabG3 and NET4 are involved in the reorganisation of the vacuole in response to the perception of the pathogen elicitor, FLG22, resulting in reduction of vacuole volume, loss of turgor pressure and stomatal closure: a key defence mechanism preventing the entry of pathogens into the leaf tissue. It is hypothesised that the perception of pathogens triggers the activation of RabG3 through an unknown mechanism, resulting in the downstream regulation of actin-driven tonoplast dynamics through NET4 (Hawkins et al., 2023).

## VAP27: An Emerging Regulator of Plant Organelle Interaction and Communication

During the characterisation of the NET protein family, ER-integral VAMP-ASSOCIATED PROTEIN-27 (VAP27) was characterised as an interactor of the NETs at organelle membrane contact sites (Wang et al., 2014). Further characterisation of this protein has demonstrated it to play crucial roles in the regulation of organelle interaction and communication in plants.

### Regulation of ER-PM Contact Sites by VAP27

In all Eukaryotes, the ER is connected to the plasma membrane at ER-PM contact sites (EPCS). In mammals and yeast, the proteins that connect the two membranes are known, with characterised roles in regulating cell architecture and organelle communications (for example, calcium signalling and lipid exchange; Scorrano et al., 2019). However, until the characterisation of the plant NET3C-VAP27 protein complex, the regulation and function of ER-PM interaction was unknown in plants (Wang et al., 2014). VAP27 proteins are important for plant growth and development (Wang et al., 2016) and regulate a multitude of subcellular processes at EPCS.

**Figure 2.**
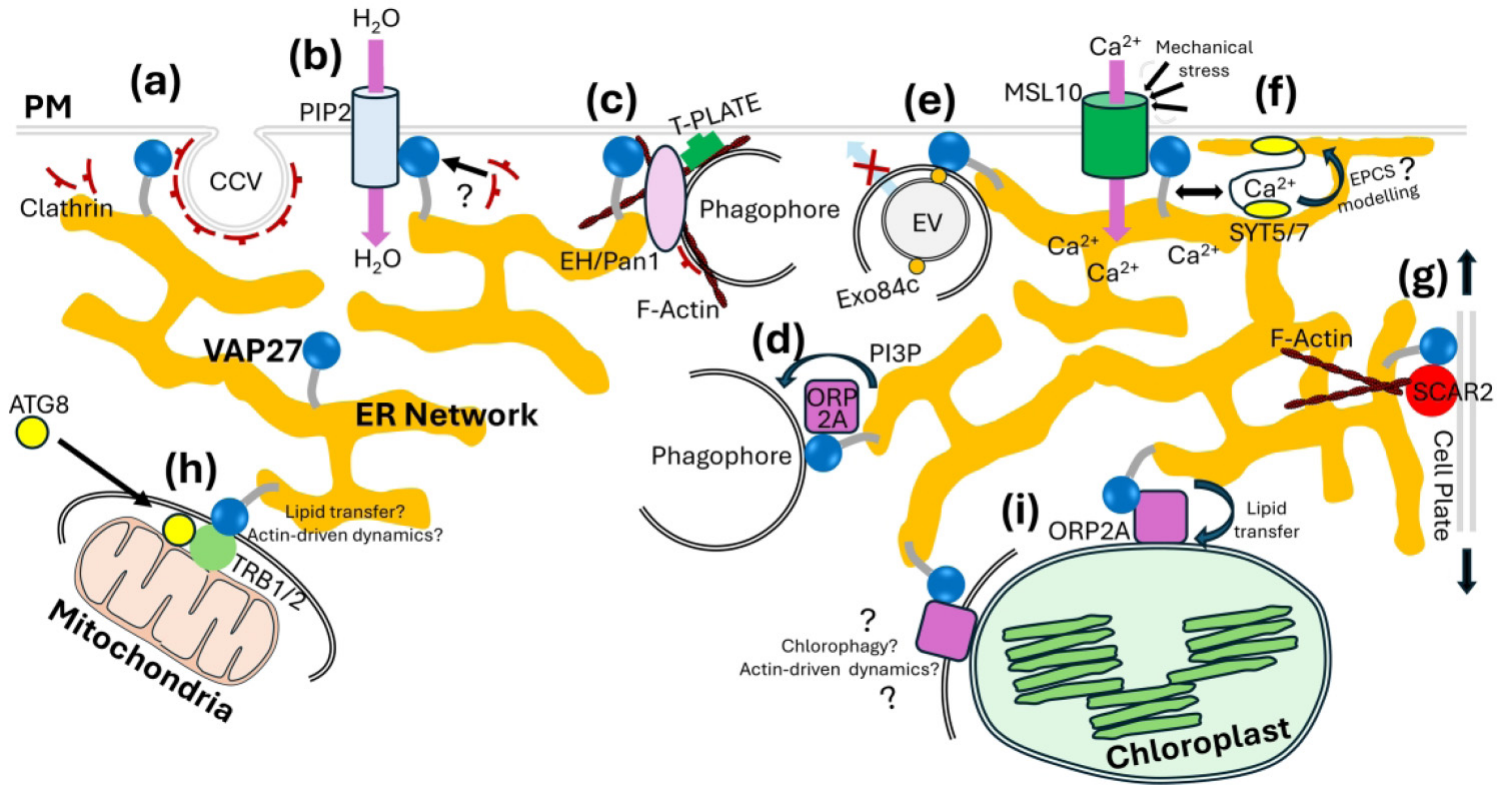
Multifaceted Functions of VAP27 at Membrane Contact Sites in Plants. (a). Endocytosis at EPCS. VAP27 interacts with clathrin to regulate the formation of clathrin coated vesicles (CCV) and endocytosis. (b). Osmotic regulation at EPCS. VAP27 interacts with PIP2-family aquaporins to modulate water influx across the PM. VAP27 may regulate internalisation of PIP2 via endocytosis, perhaps by regulating clathrin-dependent endocytosis. (c). Formation of autophagosomes at EPCS. VAP27 interacts with the actin-regulatory protein, EH/Pan1. EH/Pan1 interacts with clathrin and T-PLATE endocytic machinery. Here, VAP27 converges ER-membrane sources, PM-localised endocytic machinery and actin cytoskeleton to generate the phagophore membranes. (d). Lipid transfer at ER-Autophagosome contact sites. VAP27 interacts with the lipid-transfer protein, ORP2A, which transfers PI3P from the ER to expanding phagophore. PI3P serves as a platform for the recruitment of autophagy-regulatory proteins and the development of the autophagosome. (e). Control of exocytosis at EPCS. VAP27 interacts with the exocyst subunit, Exo84c on exocytotic vesicles (EV), likely recruiting autophagy-regulatory machinery to control their degradation via autophagy. This prevents their fusion to the PM and blocks exocytosis. (f). Calcium signalling at EPCS. VAP27 interacts with the mechanosensitive calcium channel, MSL10, which has yet uncharacterised functions in regulating EPCS structure. MSL10 is functionally linked to the EPCS structural regulators, SYT5 & SYT7. VAP27 could link MSL10 mechanosensation to downstream calcium-dependent EPCS restructuring by SYT5/7. (g). Cytokinesis at EPCS. VAP27 localises to EPCS at the expanding cell plate during cytokinesis and recruits SCAR2, the regulator of the ARP2/3 actin-nucleating complex. VAP27 and SCAR2 regulate the polymerisation of actin filaments to guide the formation of the expanding cell plate. (h). Mitophagy at EMCS. VAP27 interacts with the mitochondrial membrane proteins, TRB1/2, to tether the ER and mitochondria and drive formation of mitophagosome through recruitment of ATG8. Here, VAP27 is likely to have further roles including lipid transfer from the ER, and regulation of actin-driven membrane dynamics. (i). Lipid transfer at ER-Chloroplast Contact Sites (ECCS). VAP27 promotes ER-chloroplast association and interacts with ORP2A to transport sterols to the chloroplast from the ER. With functions in the regulation of autophagy and the cytoskeleton, VAP27 could also be involved in chlorophagy or chloroplast dynamics at ECCS.

VAP27 proteins regulate endocytosis at EPCS through interaction with Clathrin Heavy Chain and Clathrin Light Chain, and with lipids enriched at endocytic membranes (Stefano et al., 2018; Figure 2a). Loss-of-function *vap27* mutants exhibit reduced rates of endocytosis and homeostasis of endocytic membranes. It is proposed that VAP27 may be important in the recruitment of clathrin to sites of endocytosis and therefore the formation of endocytic vesicles (Stefano et al., 2018). This may be important for homeostatic mechanisms such as regulation of PM water permeability: a recent study has shown that the PM-integral aquaporin, PIP2;5 of *Zea mays* (*Zm*PIP2;5), interacts with *Zm*VAP27 (Figure 2b). The *Zm*VAP27-*Zm*PIP2;5 complex enhances cell water permeability and is implicated in regulating the endocytic internalisation of *Zm*PIP2;5 in response to salt stress (Fox et al., 2020).

VAP27 also has key roles in the regulation of autophagosome biogenesis through participating in multiple pathways. Firstly, VAP27 integrates the ER, actin cytoskeleton and PM-localised endocytic machinery to drive autophagosome biogenesis at EPCS (Wang et al., 2019). Here, VAP27 interacts with EH1/Pan1, a PM-localised activator of the ARP2/3 actin-nucleating complex, which is necessary for stress-induced autophagosome biogenesis and plant adaptation to environmental challenges including salt stress and nutrient starvation (Wang et al., 2019). At EPCS, EH1/Pan1 interacts with a number of proteins including F-actin and ARP2/3, as well as endocytic-regulatory proteins including AP2 and T-PLATE subunits, to regulate autophagic degradation of endocytic cargo (Figure 2c). The VAP27-EH1/Pan1 interaction at EPCS is a nexus of converging subcellular processes, likely encompassing supply of ER-derived membrane for autophagosome biogenesis, generation of actin-driven mechanical force for phagophore expansion, and PM-localised endocytic machinery (Wang et al., 2019).

Additionally, VAP27 has potential functions in controlling lipid redistribution at the expanding phagophore through interaction with OXYSTEROL-BINDING PROTEIN-RELATED PROTEIN 2A (ORP2A); a member of the Eukaryotic-conserved ORP-family lipid transfer proteins (Ye et al., 2022). ORP2A interacts with VAP27 and the autophagy protein, AUTOPHAGY-RELATED PROTEIN-8 (ATG8), to establish an ER-autophagosome contact site (Figure 2d). This complex is important for autophagosome biogenesis, and disruption of ORP2A expression results in defects in autophagosomal maturation and decreased levels of autophagy. ORP2A binds multiple lipids including Phosphatidylinositol-3-phosphate (PI3P) and has importance in redistributing PI3P from the ER during autophagy. During the initiation and progression of autophagosome biogenesis, PI3P accumulation is important for the recruitment of autophagy-regulatory proteins such as ATG8 to the phagophore (Nascimbeni et al., 2017; Ye et al., 2022). Disruption of ORP2A leads to the accumulation of autophagy proteins at the ER, indicating a role for the VAP27-ORP2A complex in transfer of regulatory lipids from the ER to the nascent phagophore, subsequent recruitment of autophagy proteins and autophagosomal maturation (Ye et al., 2022). Whilst this complex appears to be present at sites throughout the ER network, it is also observed at EPCS (Ye et al., 2022), potentially indicating a role for VAP27-mediated EPCS in lipid transfer.

VAP27-regulated autophagy also modulates exocytosis through coordinating turnover of exocytic vesicles (Zhang et al., 2023). VAP27 interacts with Exo84c, a plant-specific subunit of the exocyst which tethers exocytotic vesicles to the PM (Figure 2e). The interaction between VAP27 and Exo84c at ER-associated punctae is important for the targeting of Exo84c to the vacuole and its degradation via the autophagy pathway. This complex is essential for the degradation of exocyst-labelled exocytotic vesicles in Arabidopsis, with importance in regulating the senescence of stigmatic papilla cells during flowering. It is hypothesised that VAP27 may mediate a connection between the ER and PM-tethered exocyst complex at EPCS to regulate autophagy of exocytotic vesicles to achieve programmed cell death.

It is becoming apparent that VAP27-mediated EPCS are involved in calcium signalling. In animals, ER-PM contact sites are points calcium exchange between the two organelle compartments mediated by channels such as STIM1 and Orai. Furthermore, calcium signals also influence EPCS dynamics through calcium-binding EPCS structural regulators such as Extended SYNAPTOTAGMIN (E-SYT) proteins (Giordano et al., 2013). However, in plants, the involvement of EPCS in calcium signalling has not been clear, as EPCS-resident calcium signalling proteins had not been characterised. Recently, a link between EPCS and calcium signalling has been identified in plants, mediated by interaction between VAP27 and McS-Like 10 (MSL), a PM-integral mechanosensitive calcium channel (Codjoe et al., 2022; Figure 2f). MSL10 gain-of-function mutants have expanded EPCS, suggesting they may regulate calcium-dependent EPCS dynamics, perhaps in response to environmental stress. MSL10 is functionally linked to the plant E-SYT orthologues, SYNAPTOTAGMIN 5 (SYT5) and SYT7, and the expanded EPCS phenotype in *msl10* mutants is suppressed by *syt5* and *syt7* loss of function (Codjoe et al., 2022). Arabidopsis SYT proteins regulate EPCS dynamics in response to stress, and function spatially adjacent to VAP27 in order to regulate EPCS integrity (Siao et al.*,* 2016, Lee et al., 2019). VAP27-MSL10 interaction may regulate stress-responsive EPCS remodelling by functionally linking ER-structuring SYT proteins to MSL10-mediated calcium influx at the PM (Figure 2f).

Recent research also shows that VAP27 is an important regulator of cell division (Xu et al., 2025). In plants, cytokinesis occurs through the formation of a cell plate, which expands centrifugally to the cell periphery to divide the two daughter cells. The cell plate is deposited by the microtubule cytoskeleton, which is likely stabilised by actin filaments (Du et al., 2023). VAP27 localises to EPCS that become abundant at the cell plate during cytokinesis and recruits a component of the ARP2/3-activating complex, SCAR2. Loss of function of *vap27* or *scar* results in defects in root cell division including reduced division rate and polarity, likely resulting from decreased abundance of F-actin at the nascent cell plate. Here, VAP27 and SCAR regulate F-actin polymerisation at the cell plate to stabilise the microtubule cytoskeleton and guide the expansion of the cell plate in a highly ordered orientation perpendicular to root growth (Figure 2g).

### Regulation of ER-Mitochondrial Contact Sites by VAP27

Across Eukaryotes, the ER and mitochondria also physically interact at ER-mitochondrial contact sites (EMCS). In animals and fungi, EMCS have long been understood to regulate mitochondrial structure, function and metabolism, from rigorous functional analysis of the protein complexes that regulate their interaction (Scorrano et al., 2019). In plants however, the proteins that regulate ER-mitochondrial association have only recently begun to be discovered, and the functions of plant EMCS have long been unknown.

Recently, the first EMCS proteins have been characterised in plants. Proteomic screening identified the mitochondrial outer membrane protein, TraB-Family Protein 1 (TRB1), as an interactor of VAP27 (Li et al., 2022). The two proteins form a complex at the interface between the two organelle compartments to promote their association (Figure 2h). TRB1 is important for mitochondrial ultrastructure and metabolic function and has key roles in the selective targeting of mitochondria for autophagy (mitophagy). TRB1 has two conserved ATG8-interacting motif (AIM) domains which recruit ATG8 to the mitochondrial outer membrane to drive the encapsulation of mitochondria within the autophagosomal membrane (Figure 2h). VAP27 and TRB1 are therefore important for the degradation and turnover of damaged mitochondria during stress, and *vap27* and *trb* mutants accumulate dysfunctional mitochondria following mitochondrial damage (Li et al., 2022, Duckney et al., 2022b). In animals and yeast, EMCS are proposed to regulate mitophagy through the supply of ER-membrane to the mitophagosome (Bockler and Westermann, 2014). With well characterised roles in autophagy (through mediating lipid transfer, cytoskeletal regulation and recruitment of cell trafficking proteins), it is likely that VAP27 serves a key role in regulation of mitophagosome biogenesis at the ER-mitochondrial interface in plants as a regulatory nexus of these diverse subcellular processes (Duckney et al., 2024).

### Plant-Specific Roles for VAP27 at ER-Chloroplast Contact Sites

An inherently plant-specific function of VAP27 is to mediate interaction and communication between the ER and the plant-specific organelle, the chloroplast (Renna et al., 2024). VAP27 directly binds the chloroplast membrane lipid, monogalactosyldiacylglycerol (MGDG) to promote ER-chloroplast association and interacts with the lipid-transfer protein, ORP2A (Figure 2i). The ORP2A-VAP27 complex has roles in lipid transport to the chloroplast: ORP2A binds phytosterols, and *orp2a* and *vap27* mutants exhibit defects in chloroplast sterol content. Therefore, VAP27 has key roles in regulating ER-chloroplast interaction and lipid transfer, and it is likely that more functions for this protein at ER-chloroplast contact sites will emerge. With functions in the regulation of the cytoskeleton and autophagy, it is possible that VAP27 may regulate processes including chlorophagy (selective autophagy of the chloroplast), chloroplast morphology and dynamics. In agreement with this hypothesis, it has been shown that transient contact site formation between the plastid envelope and the ER are responsible for their dynamic subcellular behaviour (Mathur et al., 2023). Here, the ER serves as a site of recruitment for regulators of chloroplast fission, for example ACCUMULATION AND REPLICATION OF CHLOROPLAST 5 (ARC5) and also directly exerts physical force to separate daughter plastids (Ghosh et al., 2025). Dynamic regulation of ER-plastid tethering by VAP27 may therefore be important for the direct recruitment of regulators of chloroplast dynamics to the chloroplast membrane, supply of ER-derived regulatory lipids for recruitment of membrane-effector proteins, or recruitment of cytoskeletal regulatory proteins to the EPPC interface to drive the ER dynamics necassery for plastid division.

### Summary

The unfolding story of NET and VAP27 proteins continues to reveal novel mechanisms by which plant cells are structured. The connection between the actin cytoskeleton and membrane organelles mediated by the NET proteins are involved in the dynamic regulation of subcellular architecture and organisation. Their importance to cell growth, morphology and environmental responses are now becoming apparent. The discovery and characterisation of VAP27 as a regulator of organelle contact sites has revealed its roles in organelle tethering, structure and function, as well as inter-compartmental crosstalk and signalling. At organelle interfaces, VAP27 integrates with many subcellular machineries including regulators of the cytoskeleton, autophagy, lipid and calcium signalling, as well as cell trafficking. Future research on the NET and VAP27 proteins will continue to advance our understanding of the molecular components underpinning plant cell structure and function.
